# The Inner Nuclear Membrane Protein Src1 Is Required for Stable Post-Mitotic Progression into G1 in *Aspergillus nidulans*

**DOI:** 10.1371/journal.pone.0132489

**Published:** 2015-07-06

**Authors:** Hui-Lin Liu, Aysha H. Osmani, Stephen A. Osmani

**Affiliations:** Department of Molecular Genetics, Ohio State University, Columbus, Ohio 43210, United States of America; Florida State University, UNITED STATES

## Abstract

How membranes and associated proteins of the nuclear envelope (NE) are assembled specifically and inclusively around segregated genomes during exit from mitosis is incompletely understood. Inner nuclear membrane (INM) proteins play key roles by providing links between DNA and the NE. In this study we have investigated the highly conserved INM protein Src1 in *Aspergillus nidulans* and have uncovered a novel cell cycle response during post mitotic formation of G1 nuclei. Live cell imaging indicates Src1 could have roles during mitotic exit as it preferentially locates to the NE abscission points during nucleokinesis and to the NE surrounding forming daughter G1 nuclei. Deletion analysis further supported this idea revealing that although Src1 is not required for interphase progression or mitosis it is required for stable post-mitotic G1 nuclear formation. This conclusion is based upon the observation that in the absence of Src1 newly formed G1 nuclei are structurally unstable and immediately undergo architectural modifications typical of mitosis. These changes include NPC modifications that stop nuclear transport as well as disassembly of nucleoli. More intriguingly, the newly generated G1 nuclei then cycle between mitotic- and interphase-like states. The findings indicate that defects in post-mitotic G1 nuclear formation caused by lack of Src1 promote repeated failed attempts to generate stable G1 nuclei. To explain this unexpected phenotype we suggest a type of regulation that promotes repetition of defective cell cycle transitions rather than preventing progression past the defective cell cycle transition. We suggest the term “reboot regulation” to define this mode of cell cycle regulation. The findings are discussed in relationship to recent studies showing the Cdk1 master oscillator can entrain subservient oscillators that when uncoupled cause cell cycle transitions to be repeated.

## Introduction

The nucleus is a highly structured organelle that partitions the eukaryotic genome within the nuclear envelope (NE) which consists of an inner nuclear membrane (INM) and an outer nuclear membrane (ONM) [1]. Transport between the nuclear and cytoplasmic compartments occurs through nuclear pore complexes (NPCs), huge protein assemblies studded throughout the NE at junctions of the INM and ONMs [2–5]. Inside the nucleus, proteins and chromatin are organized into functional subdomains to modulate gene expression [6–8]. One of the most prominent nuclear subdomains is the nucleolus where rRNA is transcribed and ribosomes are assembled [9]. Although overall nuclear structure is conserved, nuclei of different species can behave very differently during mitosis [10, 11]. During closed mitoses, nuclear structures and chromatin are segregated within an intact NE while during open mitosis all nuclear structures, including the NE, nucleolus, and NPCs are disassembled. At the end of mitosis, these disassembled nuclear structures need to be reassembled to form functional G1 nuclei through regulated protein-protein, protein-lipid, and protein-nucleic acid interactions [12, 13]. In between these two extremes, the semi-open mitosis of the filamentous fungus *Aspergillus nidulans* (S1 Fig) involves partial NPC disassembly and complete disassembly of the nucleolus while the NE remains largely intact [14–17]. During mitotic exit into G1, NPCs and the nucleolus are reassembled to re-form G1 daughter nuclei [16, 17] (S1 Fig). How proteins and membranes are coordinately reassembled around segregated DNA to form functional G1 nuclei following mitosis remains poorly understood.

NPCs have been reported to form pre-pore structures on chromatin before NE reassembly [18–23]. However, depletion of the components of the pre-pore complex (vNup107-160 complex) blocks NPC assembly but does not prevent NE formation around chromatin [21, 24, 25]. This suggests that although NPC and NE assembly are normally highly coordinated processes, NE assembly can occur without major core components of the NPC. Consistent with this, it has recently been reported that the post-mitotic NE assembles directly from the mitotic ER followed by insertion of NPCs [26].

Chromatin is organized in interphase within the NE through its associations with NPCs, peripheral INM proteins, and integral INM proteins. Such chromatin-associated proteins also play roles during post-mitotic NE reassembly [1, 11]. Recent bioinformatics and proteomic studies have revealed that many INM proteins can bind chromatin and DNA suggesting that such proteins may help ensure the coordinated assembly of membrane and chromatin during nuclear formation after mitosis [18, 19, 27–29]. Live cell imaging of NE assembly in mammalian cells has also indicated that NE assembly initiates via the associations of chromatin-binding NE proteins at the ends of endoplasmic reticulum (ER) tubules followed by the flattening of tubule membrane to form a sealed and transport competent nucleus [18, 19]. In fact the roles of chromatin binding proteins in the reassembly of the functional NE during mitotic exit seem to involve functionally distinct membrane proteins acting in a redundant manner [30].

**Fig 1:**
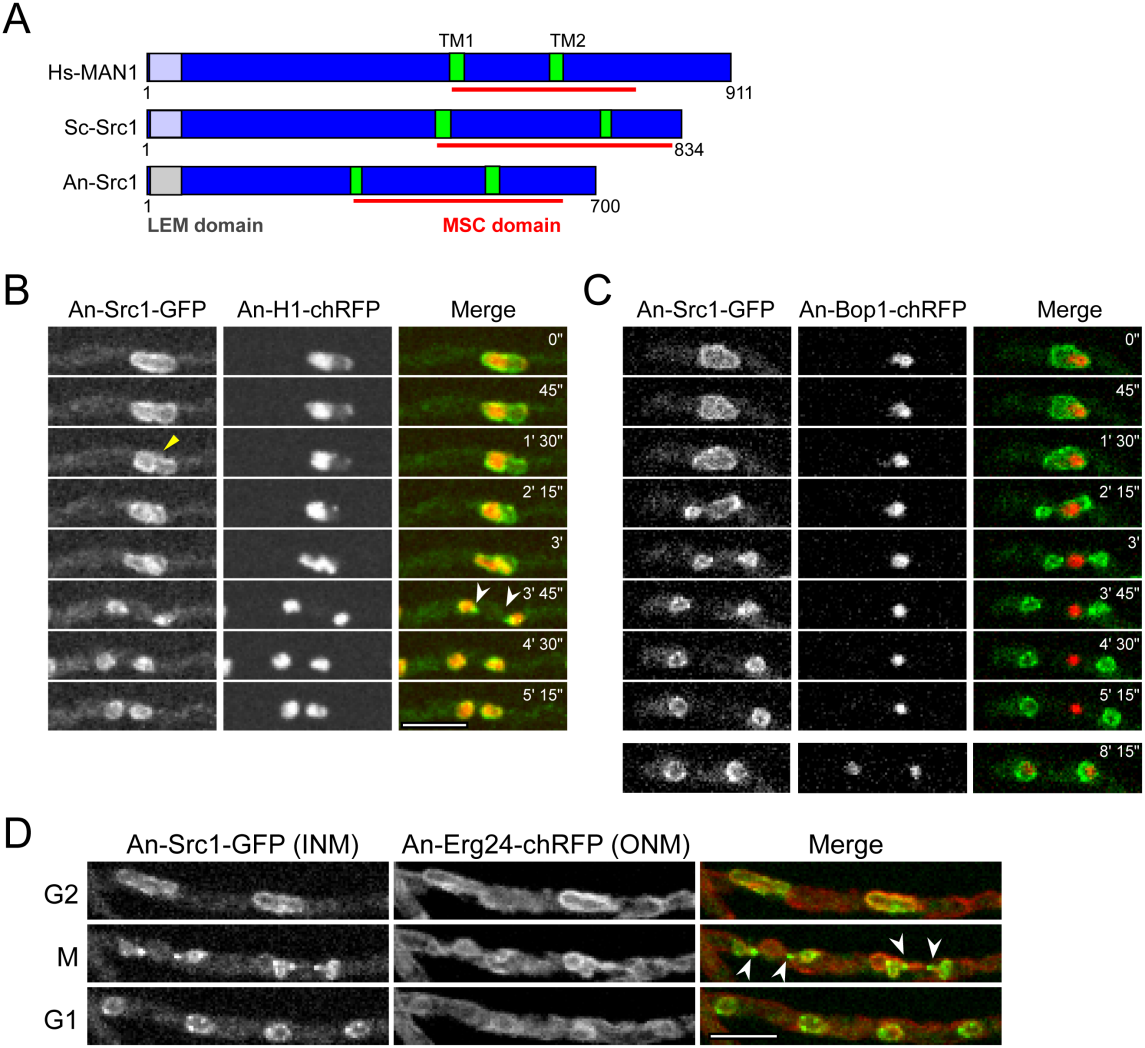
Src1 concentrates to chromatin during mitotic exit. (A) Protein domain structure of human MAN1, budding yeast Src1 (also named Heh1) compared to *A*. *nidulans* Src1 indicting the relative positions of the N-terminal LEM domain PF12949, the internal Man1-Src1p-C-terminal (MSC) domain PF09402, and the predicted transmembrane domains. (B-D) Images from live cell spinning disc confocal microscopy of mitosis in cells expressing the indicated tagged proteins. (B) Src1 associates around mitotic chromatin (marked by histone H1-chRFP) in a punctate pattern and also to two foci (white arrowheads 3’ 45”) at sites corresponding to NE abscissions during anaphase-telophase. (C) Src1 locates preferentially to the chromatin of reforming daughter nuclei in a punctate pattern and not around the nucleolus as marked by the nucleolar protein Bop1-chRFP. (D) Two nuclei shown at G2, M, and G1. The ER/NE marker Erg24 locates around both forming daughter nuclei and the nucleolus during anaphase-telophase [17]. However, at this stage of mitosis Src1 preferentially locates around the reforming daughter nuclei in a punctate pattern and not around the nucleolus (merged mitotic panel). Src1 also concentrates at the two NE abscission points indicated by the pairs of arrowheads. Scale bar 5μm.

Interestingly, many INM proteins, including lamins and the lamin-associated proteins Emerin, Otefin, BAF, Lap1 and Lap2, are only found in metazoans [31, 32]. On the other hand the LEM2/MAN1-like group of the LEM (Lap2-Emerin-MAN1) family of integral inner nuclear membrane proteins is conserved [33]. All LEM2/MAN1-like proteins contain two transmembrane domains and a MSC (MAN1/Src1p/C-terminal) motif (see Fig 1A) and help mediate NE-chromatin interactions. For example, the Man1 homolog in *S*. *cerevisiae*, Src1 (also termed Heh1) is an INM protein that preferentially interacts with subtelomeric chromatin and the ribosomal DNA (rDNA) locus [34–36] as also found for Man1 in *S*. *pombe* [37, 38]. Src1 also plays a role in sister chromatid segregation during budding yeast mitosis [39] and is involved in the assembly of NPCs, potentially through the formation of a luminal bridge with the transmembrane Nup Pom152 [40]. In mammalian cells MAN1 has been implicated in a redundant manner with other INM chromatin binding proteins during nuclear reassembly after mitosis [30]. In *C*. *elegans* the LEM domain proteins Ce-Man1 and Ce-Emerin are required for successful exit from mitosis and in their absence lagging chromosome bridges are formed which fail to lose their mitotic characteristics [41]. Interestingly in the fission yeast *S*. *japonicus*, which partially disassembles its NE during mitosis [42, 43], Man1 is required as a tether for even distribution of NPCs to daughter G1 nuclei by linking NPCs to segregating chromatin and is required for normal disassembly and inheritance of nucleoli [44].

Here, we address the roles of the INM protein Src1 during *A*. *nidulans* mitosis and early G1 and demonstrate that it is critical not for the assembly of transport competent G1 nuclei but to maintain interphase G1 nuclear architecture. The data suggest the existence of a system that monitors the generation of functional G1 nuclei which, when activated due to lack of Src1, promotes nuclei into a mitotic-like state for further attempts at error correction rather than preventing further cell cycle progression. We propose the term “reboot regulation” for this mode of cell cycle response, which promotes further attempts, rather than more time, for cell cycle error correction.

## Materials and Methods

### General techniques and image acquisition and analysis

Media, general techniques for culture of *A*. *nidulans*, western analysis, transformation, immunofluorescence, DAPI staining, and confocal microscopy imaging were as previously described [15]. Strains used in this study are listed in S1 Table. Microscopy was completed and analyzed as previously described [16].

### Growth rate analysis

Conidia from wildtype or *Δsrc1* cells were spread inoculated on plates and incubated at 23.5°C. 3–8 fields were randomly selected for imaging at 12, 15, 18, 21, and 24 hours of incubation. Germling size was measured using Photoshop software (Adobe Systems Incorporated, San Jose, CA) calibrated using a micrometer. To analyze terminal phenotypes of *Δsrc1* cells, conidia collected from heterokaryons were geminated in selective media at 23.5°C for 18 hours. 8–10 fields were randomly selected for imaging. To analyze M-G1 oscillations, cells were first germinated in minimal medium at 23.5°C for 14 hours and treated with 2.4 ug/ml benomyl to depolymerize microtubules to prevent nuclear movement within the cell. Imaging was initiated immediately after benomyl addition using one minute intervals between image acquisitions. To analyze oscillation dynamics, kymographs of indicated areas were generated using ImageJ freeware (NIH; http://rsb.info.nih.gov/ij/).

### Cell synchronization

To study phenotypes of *Δsrc1* cells during the first interphase, cells were germinated in the presence of 10 mM Hydroxyurea to prevent them from entering mitosis. To study phenotypes of Δ*src1* cells during the first mitosis, cells bearing the *nimT23* ^*ts*^ allele [45] were used to synchronize cells in late G2 by incubating at restrictive temperature (42°C) for 6 hours and 30 minutes. Following the arrest, cells were released from the arrest by replacing with room temperature (23.5°C) media and were immediately imaged.

## Results

### Localization of Src1 during telophase suggests roles during mitotic exit

Generation of daughter nuclei during exit from mitosis in *A*. *nidulans* involves restrictions and subsequent abscission of the NE at two points (S1 Fig). This process generates two daughter nuclei and a central structure containing the nucleolus [17]. Notably the NE surrounding the nucleolus during telophase has different characteristics to the NE of the forming daughter nuclei as it largely lacks core NPC proteins (Nups). One highly conserved family of integral proteins of the inner nuclear membrane includes vertebrate Man1 and related proteins (budding yeast Src1, also called Heh1). Orthologues of Src1 contain two transmembrane domains, an N-terminal HeH/LEM domain (PF12949) and an internal Man1-Src1p-C-terminal domain (MSC, PF09402) [32, 33]. One such protein (AN3910) has been defined by Mans et al., [32] and also at the *Aspergillus* Genome Database site (AspGD) (http://www.aspergillusgenome.org/cgi-bin/locus.pl?locus=AN3910#summaryParagraph) as the only *A*. *nidulans* orthologue of Src1 (Fig 1A). Live cell imaging of functional endogenously GFP-tagged Src1 revealed that Src1 localizes to the nuclear periphery in interphase and early mitosis. However, during anaphase/telophase, Src1 concentrates around the condensed chromatin of the forming daughter nuclei with little apparent at the central NE located around the nucleolus (Fig 1B 3’ 45’). Of note, two foci were also seen at the restriction points at which the double abscission of the NE would be expected to be occurring during telophase (Fig 1B, indicated by white arrowheads). These foci were confirmed to coincide with the abscission sites of the nuclear envelope by comparing the location of the ER/ONM protein Erg24 to that of Src1 (Fig 1D) during the double NE abscission step (S1 Fig) of telophase [16, 17]. This comparison also confirmed that Src1 concentrates with the NE associated with chromatin but not the NE surrounding the nucleolus during mitosis. To more directly show Src1 did not locate at the NE around the nucleolus during mitotic exit we followed Src1-GFP and the nucleolar protein Bop1-chRFP (Fig 1C). Although Src1 located around the entire nucleus during interphase and up to metaphase of mitosis, at anaphase/telophase Src1 preferentially associated with forming daughter nuclei and did not locate around the nucleolus (Fig 1C, compare time 2’15” to 3’). Collectively the data suggest a differential segregation of an ONM protein and Src1 during anaphase/telophase; the ONM protein located around both daughter nuclei and the nucleolus while Src1 locates preferentially around forming daughter nuclei and not the nucleolus during telophase. Additionally Src1 also concentrates at the two NE abscission sites during generation of daughter nuclei.

**Fig 2:**
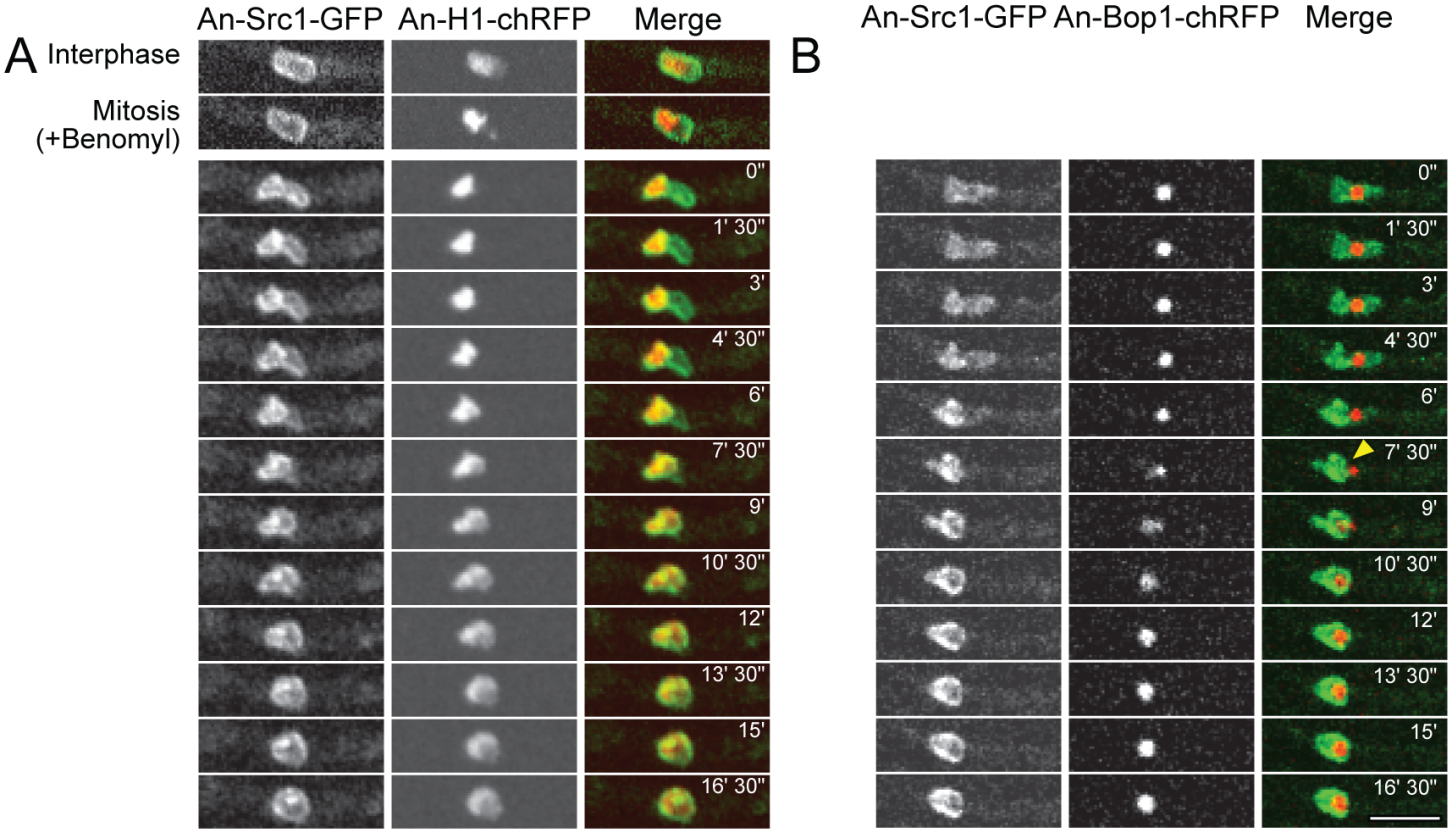
Src1 preferentially locates around chromatin during mitotic exit in the absence of mitotic spindle function. Cells expressing the indicated pairs of tagged protein were treated with the microtubule poison benomyl and imaged during spindle independent mitotic exit (SIME) [17]. (A) Time-lapse images of Src1-GFP with the DNA marker H1-chRFP during the transition from a SAC imposed mitotic arrest and SIME, the start of which is indicated as time 0. Src1 can be observed to retract from the nuclear region to the right and locate preferentially around the chromatin located in the left side of the nucleus. When this process is completed (time 7’30”) the chromatin begins to undergo decondensation. (B) A similar time course as A but following the nucleolar marker Bop1 and Src1 to show that it is the nucleolus from which Src1 retracts when it preferentially locates to mitotic chromatin during mitotic exit. The arrow at time 7’ 30” indicates completion of the removal of Src1 from around the nucleolus. Scale bar 5μm.

In *A*. *nidulans* several mitotic events can occur in the absence of the mitotic spindle including exclusion of the nucleolus from the nucleus, its subsequent disassembly in the cytoplasm, and reassembly within nuclei [17, 46]. During such spindle independent mitotic exit (SIME) core NPC proteins, including Nup170, relocate from around the entire nuclear periphery to preferentially locate around the mitotic chromatin leaving the nucleolus outside the nucleus. Dispersed peripheral NPC proteins, such as Nup49, are also preferentially recruited back to the NE surrounding chromatin during SIME. We therefore determined if Src1 might similarly display a preferential redistribution to chromatin during SIME. During mitotic arrest, due to spindle assembly checkpoint (SAC) activation (entry into mitosis after addition of benomyl to depolymerize microtubules), chromatin condensed and Src1 remained around the nuclear periphery, surrounding both the condensed chromatin and the nucleolus (Fig 2A, compare the Interphase and Mitosis +Benomyl panels). After a defined period of time (~45 minutes) the SAC is inactivated in a regulated manner allowing exit from mitosis in the absence of spindle function [46]. During exit from SAC arrest there was a clear transition in the location of Src1; it retracts from the region of the nuclear periphery not surrounding chromatin to locate more exclusively around mitotic chromatin (Fig 2A, compare merged image 0” to 7’30” and time points in between). After Src1 locates around the condensed chromatin, the chromatin then undergoes decondensation indicating exit from the mitotic state (Fig 2A 7’30”– 13’30”).

Retraction of Src1 from around the nucleolus to around chromatin during SIME was then directly observed by following Scr1 and the nucleolar marker Bop1 (Fig 2B). During mitotic SAC arrest, Src1 encloses the whole nucleus including Bop1. However, during mitotic exit Src1 moved to preferentially encompass the nuclear region that does not contain Bop1 (Fig 2B, indicated by a yellow arrowhead). Bop1 was then re-imported to the transport competent G1 nucleus to help reestablish its nucleolus. The findings indicate Src1 behaves like core NPC proteins during exit from mitosis into G1 and displays a preferential association around chromatin.

### Src1 is essential

**Fig 3:**
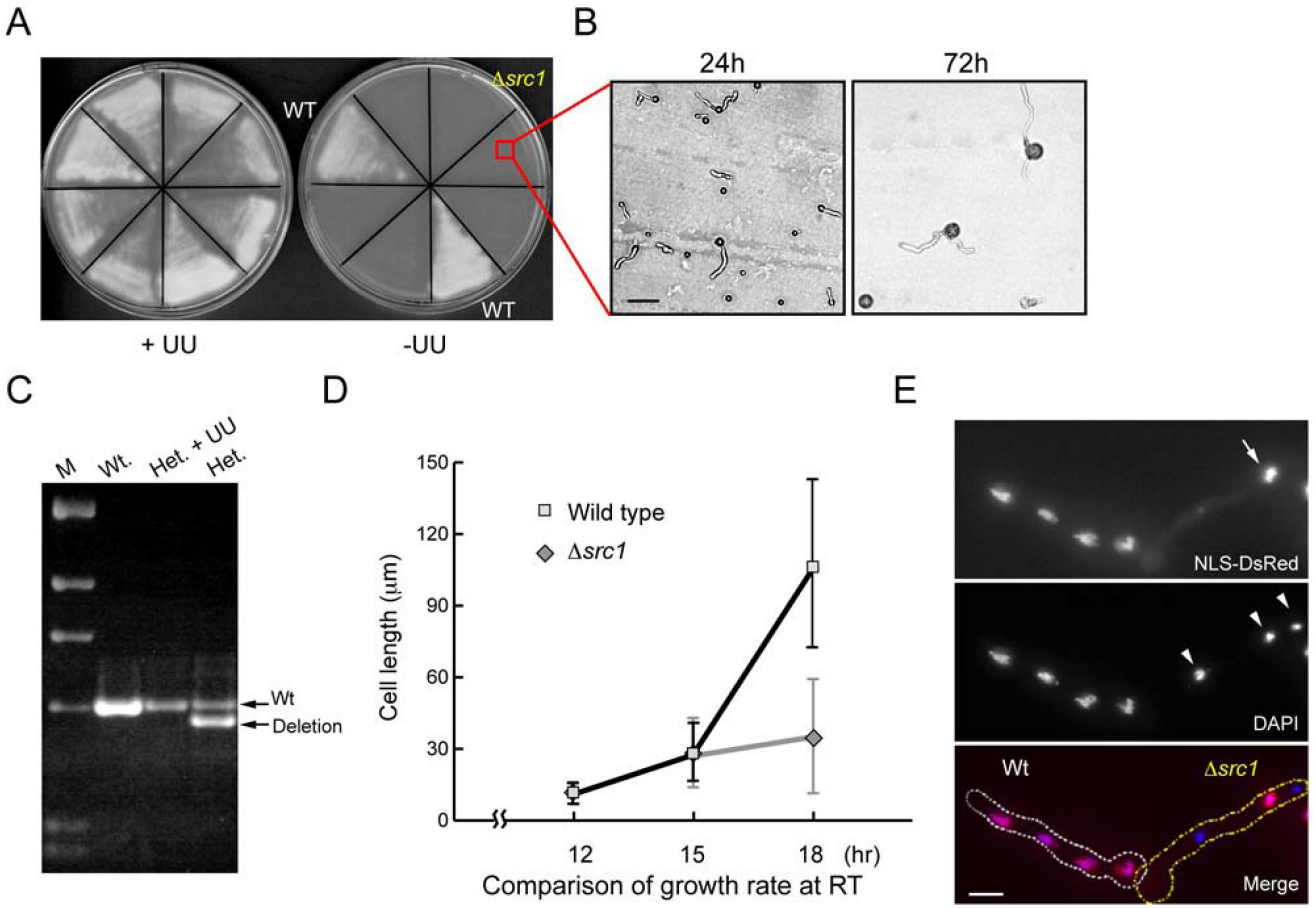
Src1 is essential. (A) Conidia (asexual spores) from wildtype control (*pyrG* ^+^) *or* Δ*src1*::*pyrG* deletion primary transformants were replica streaked onto nonselective (+UU) and selective (-UU) plates and grown for 40 hours for photography. Conidia that are not able to grow on–UU media are from primary Δ*src1*::*pyrG* / *src1 pyrG*89 heterokaryon transformants indicating *src1* to be essential. (B) Bright field image of the limited growth of conidia from *Δsrc1*::*pyrG* / *src1 pyrG*89 heterokaryons on selective media for 24 and 72 h. Scale bar 10 μm. (C) Diagnostic PCR using DNA isolated from a wild-type strain (Wt.), from a putative heterokaryon grown in +UU media (stops selection for *Δsrc1*), and from a putative heterokaryon growing in-UU selective media. The deletion allele carried in the heterokaryotic transformant is rapidly lost upon propagation without selection indicating the transformant is not a diploid but a heterokaryon. (D) The degree of growth of both wildtype and *Δsrc1* cells were compared at 12, 15, and 18 hours at 23.5°C by measuring the average length of germlings. (E) *Δsrc1* germlings fixed and stained with DAPI to reveal nuclear defects, including condensed DNA, odd numbers of nuclei (indicated by white arrowheads), and an unusual pattern of nuclear transport evidenced by only one nucleus being able to accumulate NLS-DsRed (indicated by arrow). Scale bar 5 μm.

We replaced the coding region of *src1* with the *pyrG* nutritional marker to determine the null phenotype. Using heterokaryon rescue [47] we determined that *src1* is essential (Fig 3A and 3C) and after *src1* deletion heterokaryons were generated that had two types of genetically distinct nuclei; nuclei with Src1 intact but which are *pyrG* ^**-**^ and nuclei that have Src1 deleted (Δ*Src1*) and are *pyrG* ^**+**^ (Fig 3C). Both nuclear types are therefore required for heterokaryotic growth when selection for *pyrG* ^+^ is imposed. Because the asexual spores generated from heterokaryons contain a single nucleus, the *src1* + *pyrG* ^**-**^ nuclei and Δ*src1* + *pyrG* ^**+**^ nuclei are segregated into individual spores. By germinating spores from the heterokaryon on medium selective for *pyrG* ^**+**^ (hence Δ*Src1*) we determined that cells lacking *Src1* are able to break dormancy and undergo polarized growth at a comparable rate to normal cells during the first 15 hours of their growth (Fig 3D). The Δ*src1* cells failed to continue normal growth resulting in a terminal arrest phenotype as germling cells (Fig 3B). Visualization of nuclei after DAPI staining showed that a significant percentage of *Δsrc1* cells (52%) contained odd numbers of nuclei (Fig 3E). This suggests that some nuclei had undergone mitotic division while others had not, something rarely seen in wildtype cells in which the nuclear number doubles during each para-synchronous mitosis. In addition, *Δsrc1* nuclei often contained condensed chromatin of uneven intensity (data not shown) suggestive of mitotic DNA segregation defects.

To further investigate the defects caused by *Src1* deletion, we performed the deletion analysis in strains expressing the nuclear transport marker NLS-DsRed and also followed nuclear number by DAPI staining fixed cells. During interphase NLS-DsRed is transported into nuclei. During mitosis NPCs undergo partial disassembly, nuclear pores are opened, and NLS-DsRed is dispersed throughout the cell [14, 48]. Upon mitotic exit, NPCs are reassembled and active nuclear transport resumes importing NLS-DsRed back into G1 nuclei. Because cytokinesis does not occur during the early stages of spore germination and all nuclei in *A*. *nidulans* cells traverse the cell cycle synchronously [49], NLS-DsRed is typically within all nuclei of multinucleated cells during interphase and dispersed from all nuclei during mitosis. Strikingly however, we observed *Δsrc1* cells in which some nuclei were transport competent whilst other nuclei in the same cytoplasm did not import NLS-DsRed (Fig 3E, note only one of the three nuclei defined by DAPI staining is transport competent in the *Δsrc1* cell).

### Src1 is not required for functional NPC formation

**Fig 4:**
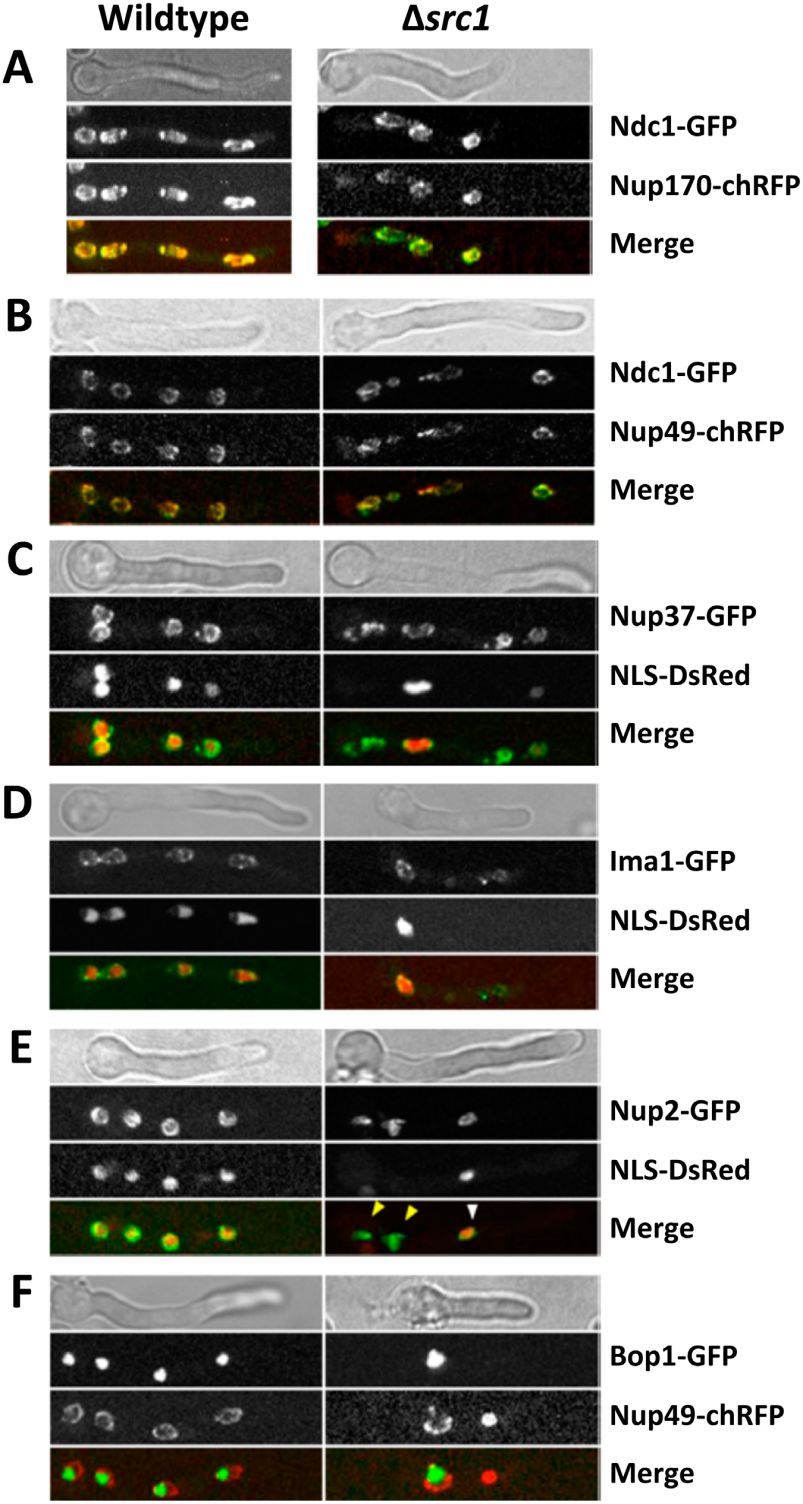
Deletion of Src1 causes pleotropic nuclear defects. (A-F) Endogenously GFP-tagged or chRFP-tagged nuclear proteins were analyzed in both wildtype and *Δsrc1* cells germinated at 23.5°C for 18 hrs to characterize the terminal arrest phenotypes. For each panel representative cells are displayed as indicated. (A and B) The transmembrane Ndc1, core Nup170 and peripheral Nup49 Nups all locate around the nuclear periphery in both Wt and *Δsrc1* nuclei indicating normal NPC assembly. (C) Although the core Nup37 protein locates around all four nuclei in the Δ*src1* cell only one nucleus is highly active for nuclear transport of NLS-DsRed. (D) Ima1 locates around the NE in both the Wt and Δ*src1* cells although only one nucleus is transporting NLS-DsRed in the Δ*src1* cell. (E) Nup2 localizes to the nuclear periphery of the transport competent interphase nuclei in the Wt cell. However in the Δ*src1* cell only one nucleus, indicated by a white arrowhead, localizes Nup2 to its nuclear periphery and is transport competent while in the other nuclei (indicated by yellow arrowheads) Nup2 locates to the nuclear interior, potentially at chromatin, and are more mitotic-like. (F) The nucleolar marker Bop1 locates within all Wt nuclei but is not imported to both nuclei of the Δ*src1* cell. In the nucleus that does not contain any Bop1 the Nup49 protein locates within the nucleus potentially at chromatin; a location only seen during mitotic exit in Wt cells.

We determined the location of Ndc1 (core transmembrane Nup), Nup170 (core Nup), and Nup49 (peripheral Nup) to see if their recruitment to NPCs was defective in the absence of Src1. The distribution of these proteins was analyzed in *Δsrc1* spores germinated for 18 hours. As demonstrated in the representative images shown in Fig 4A and 4B, Src1 is not required for the NE recruitment of Ndc1, Nup170 or Nup49 which were all seen to localize around the nuclear periphery in both wildtype and *Δsrc1* cells. Notably however, *Δsrc1* nuclei often displayed an irregular size and shape when compared to controls (Fig 4A and 4B).

We next asked if the distribution of NPC or NE proteins was changed in nuclei that were not transport competent in the absence of Src1. The core NPC component Nup37, as well as the orthologue of fission yeast inner nuclear membrane protein Ima1 (AN5735, S2 Fig) were found to locate at the nuclear periphery in nuclei that were, or were not, transporting NLS-DsRed (Fig 4C and 4D).

Nup2 has unique cell cycle specific locations [15] [50]. During interphase Nup2 locates around the nuclear periphery (Fig 4E Wildtype) but during mitosis it all locates to chromatin (not shown, see [15]). In some cells lacking Src1 we could identify nuclei that had Nup2 located around their nuclear periphery (Fig 4E, white arrowhead) although it was not located around the periphery of other nuclei within the same cell (Fig 4E, yellow arrowheads). Notably the nuclei that had Nup2 located around their periphery were transport competent and apparently in interphase of the cell cycle (Fig 4E, indicated by a white arrowhead) while those nuclei that had Nup2 associated more interior to nuclei (perhaps on their chromatin) were not transport competent and therefore resembled mitotic nuclei (Fig 4E, indicated by yellow arrowheads). These observations indicate that nuclei within the same *Δsrc1* cell can exist in different morphological states with regards to their cell cycle characteristics.

The generation of new nucleoli after mitosis in *A*. *nidulans* is dependent upon nuclear transport being reestablished in both daughter G1 nuclei providing them equal ability to import nucleolar proteins released from the parent nucleolus as it disassembles in the cytoplasm [17]. However, tracking GFP-tagged Bop1, a nucleolar marker, in Δ*src1* cells revealed a range of defects in nucleolar segregation including cells in which one nucleus contained Bop1 next to one in which no Bop1 could be detected (Fig 4F). It was also noticeable that in nuclei not containing Bop1, the NPC marker Nup49 did not locate around the nuclear periphery but located within the nucleus as if associated with chromatin (Fig 4F Δ*src1*, nucleus to the right). The only phase of the cell cycle during which Nup49 appears associated with the nuclear interior is during exit from mitosis. Therefore the observations suggest that nuclei without Bop1 might not have exited mitosis correctly or that they had exited mitosis but subsequently attained a more mitotic-like state (perhaps fanciful, but see below).

### After mitosis, G1 nuclei of cells lacking *src1* can attain a mitotic-like morphology

**Fig 5:**
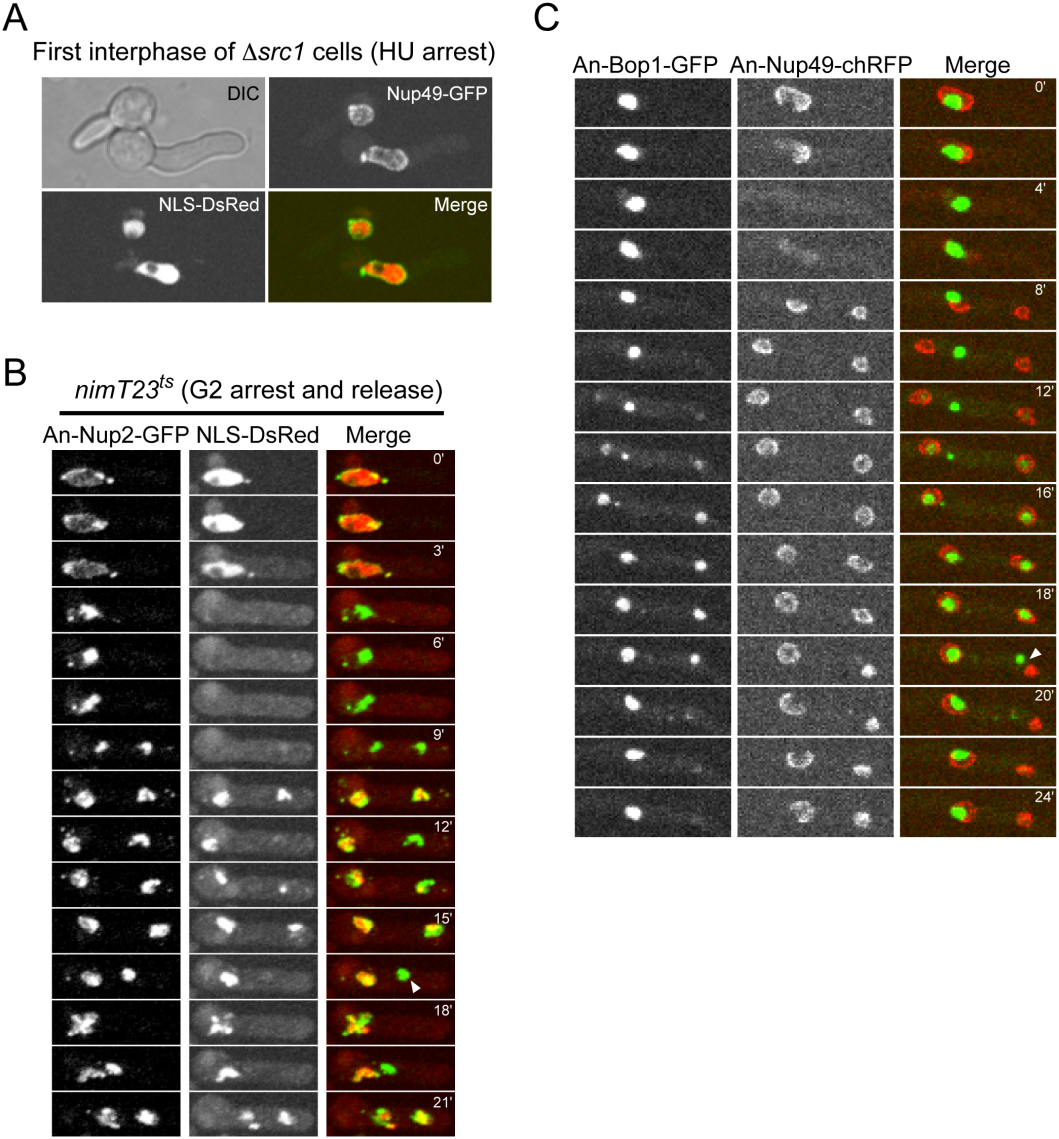
*Δsrc1* nuclei undergo architectural modifications from G1 that are typical of mitosis. (A) *Δsrc1* spores were germinated in the presence of 10 mM HU to arrest them in interphase. NLS-DsRed was transported within the nucleus in *Δsrc1* cells and Nup49 located around their nuclear periphery as typical of interphase Wt cells. (B-C) The localization of Nup2, NLS-DsRed, Bop1, and Nup49 were monitored in the first mitosis of *Δsrc1* cells after release from G2 arrest imposed by the *nimT23* ^ts^ allele. (B) As occurs during normal mitosis, Nup2-GFP translocates to condensed chromatin and NLS-DsRed escapes from the nucleus during mitosis in Δ*src1* cells. However, soon after the two G1 nuclei are established and accumulate NLS-DsRed, with Nup2 now around their nuclear periphery, the nucleus indicated by the white arrowhead becomes mitotic-like with Nup2 locating to chromatin and losing its nuclear transport capacity. These effects were then largely reversed at the 21’ time point with the nucleus re-importing NLS-DsRed again. (C) As occurs during normal mitosis, Nup49 disperses from NPCs and returns to NPCs of the two new G1 nuclei generated during mitosis (0–8’). The nucleolus, marked by Bop1-GFP, then disassembles with the released nucleolar proteins being reimported into the new G1 nuclei. This process occurs normally in the absence of Src1 and is completed apparently normally (time point 17’). However the G1 nuclei are not normal as the nucleus to the right undergoes transitions typical of mitosis including the movement of Nup49 into the nucleus, presumably onto chromatin, and the disassembly of Bop1 after it is apparently expelled to the cytoplasm (indicated by a white arrowhead). This nucleolus then disassembles and Bop1 is imported into the transport competent daughter nucleus.

To define the role of mitosis in generating Δ*src1* transport incompetent nuclei we germinated Δs*rc1* spores from heterokaryons in the presence of hydroxyurea (HU), an inhibitor of DNA replication, to arrest them in interphase before their first mitosis [51]. Δ*src1* spores germinated in HU were able to transport NLS-DsRed into their single interphase nuclei (Fig 5A). We also arrested Δ*src1* cells in late G2 before entry into mitosis using the *nimT23* ^*ts*^ mutation that prevents activation of Cdk1 [45]. At the G2 arrest point of *nimT23* ^*ts*^ *Δsrc1* nuclei were transport competent (example in Fig 5B, 0’). These results indicate that nuclear transport is active in *Δsrc1* cells before they undergo mitosis and that generation of transport incompetent nuclei depends upon passage through mitosis. To investigate this we followed the distribution of Nup2 and NLS-DsRed during the first cell cycle in *Δsrc1* cells released from the *nimT23* G2 arrest into mitosis. During entry into mitosis Nup2 moved from the nuclear periphery onto chromatin and NLS-DsRed dispersed in *Δsrc1* cells; as occurs during wildtype mitosis (Fig 5B, 3’ to 4’30”). DNA segregation then occurred and as both daughter nuclei exited mitosis Nup2 relocated back to the nuclear periphery and nuclear transport was reestablished, as expected during exit from mitosis into G1 (Fig 5B 10’ 30”). Subsequently, however, there was a defect in some G1 nuclei as they surprisingly reverted back to a more mitotic-like state indicated by the dispersal of NLS-DsRed from nuclei (Fig 5B, 16’30” nucleus indicated by arrowhead).

If, after mitosis, *Δsrc1* nuclei can change into a mitotic-like state then additional mitotic changes in such nuclei might be evident. As described above, mitotic nucleolar segregation involves expulsion of the nucleolus from nuclei into the cytoplasm followed by its disassembly and re-import of the released nucleolar proteins into daughter nuclei (S1 Fig). We therefore monitored the behavior of the Bop1-GFP nucleolar marker, and the mitotically dispersed Nup49-chRFP, in *Δsrc1* cells (Fig 5C). We observed that both Bop1 and Nup49 locate normally in G2 (Nup49 around the nuclear periphery, Bop1 occupying the nucleolar subdomain of the nucleus, Fig 5C, 0’) in the absence of *src1*. As expected, upon entry into mitosis Nup49 disassembled from NPCs and dispersed throughout the cell. Nup49 then returned to daughter nuclei during mitotic exit as the nucleolus was excluded to the cytoplasm (Fig 5C, 2’- 10’). The nucleolus then disassembled normally and Bop1 was released to the cytoplasm and transported into the forming G1 daughter nuclei to regenerate their nucleoli. Because the disassembly of the parent nucleolus occurs at the same time as daughter nucleoli are being formed, three Bop1 foci were transiently observed as expected (Fig 5C 12’– 16’). Therefore, lack of Src1 had no obvious effect on the behavior of either Nup49 or Bop1 during mitosis and early G1 and two apparently normal G1 nuclei formed (Fig 5C 18’). However, these post-mitotic Δ*src1* nuclei did not all maintain their G1 architecture as evident in the behavior of the nucleus to the right in Fig 5C after 18’. In this nucleus Nup49 undergoes an unexpected translocation from around the nuclear periphery to a focus that is separate from Bop1 (Fig 5C 19’ arrowhead). Nucleolar Bop1 then dramatically undergoes disassembly, an event that normally happens only during exit from mitosis, and the released Bop1 protein was transported into the other G1 nucleus. The result is the generation of two nuclei, one with a nucleolus, the other without (similar also to the situation shown in Fig 4F for the Δ*src1* fixed cell). The data indicate that post-mitotic G1 nuclei in cells lacking Src1 are not structurally stable and undergo morphological changes that are characteristic of mitosis. Such nuclei therefore switch from a G1 to a more mitotic-like state.

### In cells lacking Src1 nuclei oscillate between periods when nuclear transport is active and inactive after mitosis

**Fig 6:**
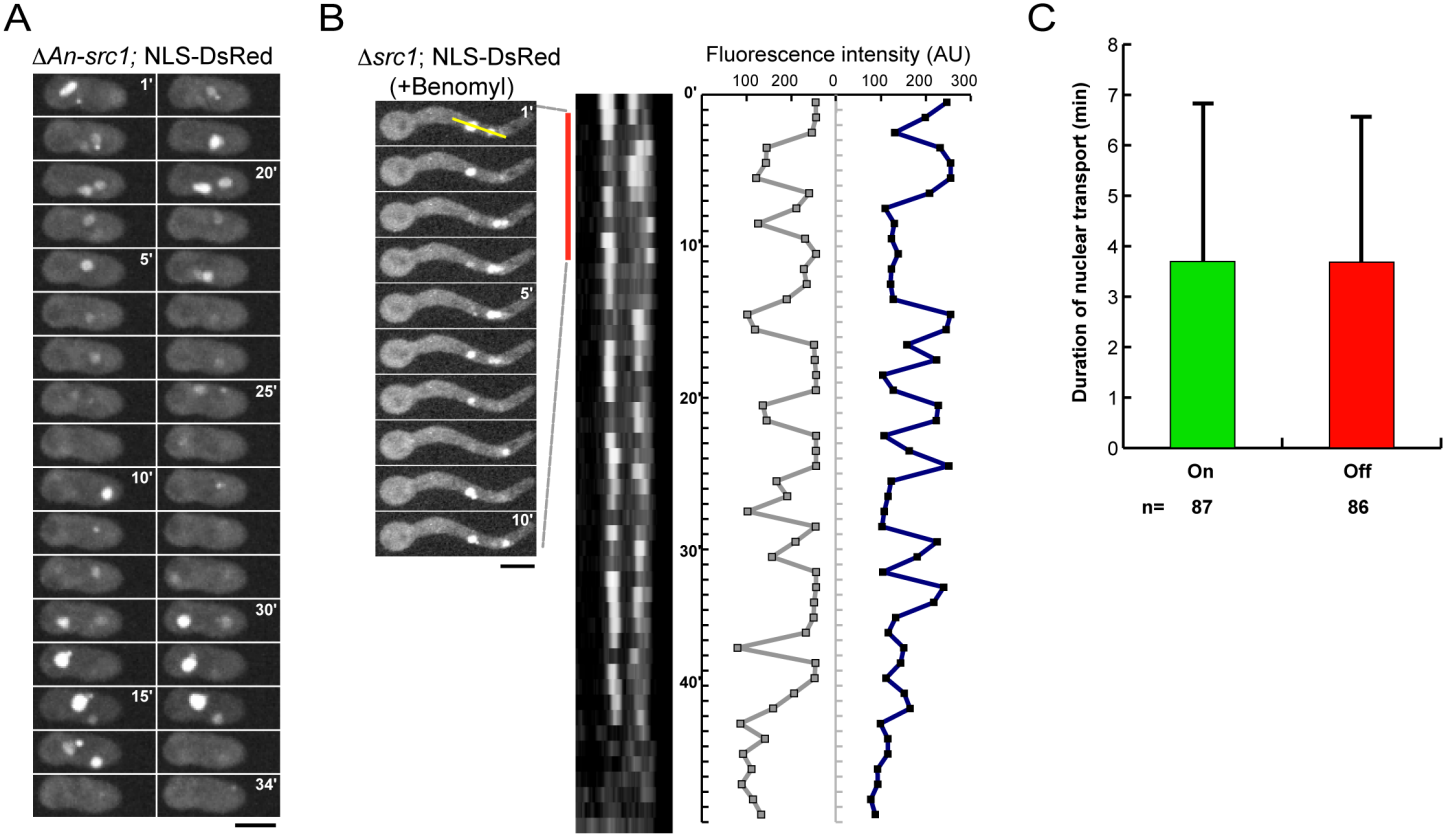
Nuclei oscillate between mitotic-like and interphase-like states in Δ*src1* cells. (A) Time course imaging over 34 minutes of NLS-DsRed in a *Δsrc1* cell indicates its nuclei oscillate between nuclear transport active and inactive states. (B) To more clearly track individual nuclei within *Δsrc1* cells the distribution of nuclear NLS-DsRed was monitored in benomyl treated cells to stop nuclear movements. Nuclei can be seen to oscillate between transport active and inactive states through the kymograph representing the intensity of NLS-DsRed across the yellow line with time; as also plotted as nuclear fluorescence intensity. The vertical red line indicates the first ten minutes of the live cell imaging. (C) Analysis of the data revealed nuclei spent on average an equal time between nuclear transport active and inactive states with no discernable pattern between each state between nuclei within individual cells.

Time lapse microscopy of NLS-DsRed in Δ*src1* cells undergoing their first cell cycle found their single nuclei constitutively imported NLS-DsRed. However, analysis of post-mitotic Δ*src1* cells containing at least two nuclei showed that nuclei of cells lacking Src1 could oscillate between active and inactive nuclear transport states (Fig 6). These studies (Fig 6A) were complicated by the movement of nuclei within cells which made identification of individual nuclei difficult. Because nuclear movement is dependent on microtubules, we depolymerized microtubules using benomyl treatment then followed NLS-DsRed in post mitotic Δ*src1* nuclei that were now more stationary (Fig 6B). The analysis revealed that nuclei behaved in a nuclear-autonomous manner and that, although variable, on average switched from a transport competent state to a transport incompetent state every 3–4 minutes without a preferential stay in either one of the states (Fig 6C). We have never seen such oscillatory behavior in wildtype cells. Because nuclei were able to oscillate between transport active and inactive states in the presence of benomyl it suggests these transitions are not regulated by the SAC or, more likely, that such nuclei have passed the SAC arrest point.

## Discussion

### The dynamic locations of Src1 suggest roles during mitotic exit

If the ONM and INM retained their respective complement of proteins, and remained as paired double membrane structures during mitosis, it would be expected that protein markers for both would display the same distribution. However, Src1 does not completely co-localize with the ER/NE marker during mitosis. Instead Src1 shows a preferential location around the chromatin of forming daughter nuclei; being less evident around the nucleolar region during late mitosis. In addition, Src1 locates at the two NE abscission points that separate daughter nuclei from the nucleolus during telophase. At this time we do not know the basis for the differential localization of the ER marker and Src1. However, given that Src1 orthologues are known to be involved in interactions between chromatin and the INM (see introduction for references), the locations we have observed support a potential role for Src1 during mitotic exit in association with chromatin.

### Lack of Src1 causes G1 defects that allow transitions typical of mitosis

Src1 is essential although spores lacking the *src1* gene were able to break dormancy and undergo early growth and germtube extension at a rate comparable to control cells. However, they were unable to maintain a normal growth rate and generated a terminal arrest phenotype as small germlings. Further analysis of the arrest phenotype of *src1* deleted cells did not reveal major defects in the ability of NPC proteins to locate to NPCs. However, analysis of fixed cells revealed that nuclei within individual cells that had gone through mitosis displayed different characteristics; some nuclei had features of mitotic nuclei (nuclear transport off) while others had interphase characteristics (nuclear transport on). Because the cell cycle is synchronous within individual *A*. *nidulans* cells, it is rare to observe mitotic nuclei and non-mitotic nuclei in the same cell. Deletion of Src1 therefore promotes nuclei to act in a nuclear-autonomous manner in the cell cycle after mitosis. This was most dramatically apparent during live cell imaging. The nuclei in cells after the first mitoses lacking Src1 oscillate in an autonomous manner switching between states of active then inactive nuclear transport. Cell cycle block-release experiments uncovered the dependence of passage through mitosis in the generation of these unexpected phenotypes. Arresting the cell cycle in S-phase or G2 showed that Src1 is not required for generation of transport competent nuclei during the first interphase of spore germination. Further, after release of G2 arrested cells into mitosis, we found that mitosis was completed without obvious defects to generate two transport competent, apparently normal, G1 nuclei. Such G1 nuclei were also capable of reforming their nucleoli, a process involving nuclear import of nucleolar proteins from the cytoplasm. However, such nuclei did not all maintain their G1 architecture but displayed mitotic-like features within minutes of first entering G1 including reversal of their capacity to transport NLS-DsRed. In addition, Nup49 was observed to move from its NPC location around the nuclear periphery during G1 in towards the nuclear interior. Most dramatically, such nuclei also expelled their nucleolus into the cytoplasm where it underwent disassembly. The disassembled nucleolar proteins were imported into the daughter G1 nucleus present in the cell. The only period during the cell cycle in which the nucleolus is dispelled into the cytoplasm is late mitosis. Similarly, the only cell cycle phase during which Nup49 associates towards the nuclear interior is at chromatin during the late stages of mitosis. This indicates that G1 nuclei formed in the absence of Src1 are unable to maintain their normal interphase morphological features but instead undergo structural transitions typical of late mitotic nuclei.

### A working hypothesis (reboot regulation) to explain why G1 nuclei lacking Src1 intermittently display morphological features of mitosis

Typically, in response to cell cycle defects, checkpoint regulatory systems act to stop further cell cycle progression until the defect is corrected [52]. However, although the defect caused by lack of Src1 affects G1 nuclei, rather than causing G1 arrest, it promotes oscillations (based upon changes in nuclear function and architecture) between G1-like and mitotic-like states. This phenotype provides evidence of a previously unrealized response to a defective cell cycle transition involving the transition becoming unstable, and repeated, rather than causing cell cycle arrest. Such a response makes biological sense if a cell cycle defect would be difficult to correct from an arrested cell cycle state. For example, it is possible that defects in daughter G1 nuclear architecture involving incorrect distribution of DNA and/or the nucleolus would be difficult to redress from a G1 arrest. Given the phenotypes caused by lack of *A*. *nidulans* Src1 and the roles its orthologues play in budding [36] and fission yeasts [44] the defects in G1 caused by lack of Src1 might involve DNA-INM interactions, nucleolar-INM interactions, DNA segregation or nucleolar segregation. We suggest that such defects in G1 nuclei would be difficult to correct from G1 and might better be dealt with by retrying the M-G1 transition and such a response is supported by our findings.

The oscillatory response we have uncovered in response to lack of Src1 involving transitions between G1 and mitotic-like states might therefore reflect repeated attempts to form normal G1 nuclei, which are unable to form in the absence of Src1. We suggest the term “reboot regulation” to describe responses to cell cycle defects that cause the defective transition to be repeated rather than causing cell cycle arrest. Reboot regulation allows further opportunities, and not just further time, to complete a cell cycle transition correctly.

The idea that a defective cell cycle transition can cause the transition to be undone and repeated, as proposed during reboot regulation, is conceptually analogous to the process by which incorrect spindle microtubule attachments to kinetochores during mitosis promotes undoing the incorrect attachments to allow further attempts at attaining correct attachments [53]. The reversal of incorrect kinetochore attachments is elegantly regulated by sensing tension across the centromere which stabilizes correct attachments, while phosphorylation of kinetochore proteins by Aurora B kinases eliminates incorrect attachments [53]. How cells detect and then promote reformation of defective G1 nuclei is likely to involve even more complex regulation which remains to be investigated. However, a recent insight to budding yeast cell cycle regulation provides hints regarding how such regulation could work. Studies have shown that a cyclin-dependent kinase (Cdk) oscillator can entrain peripheral oscillators that then control specific cell cycle events, such as mitotic nucleolar release of the Cdc14 phosphatase [54–56]. As part of these studies it was found that setting Cdk1 activity at steady non-oscillatory levels would uncouple a normally phase-locked peripheral oscillator allowing it to promote repeated endocycles of nucleolar Cdc14 release. If similar entrained peripheral oscillators exist in *A*. *nidulans* these could potentially help provide the regulatory mechanism by which absence of Src1 promotes reboot regulation and cycles between G1 and M-like states. In this scenario defects caused by lack of Src1 after G1 nuclei formation would lock a master oscillator causing uncoupling of an M-G1 peripheral regulatory oscillator. This would then allow cycles of M-G1 to occur until the defects were repaired. Once corrected the master oscillator would be released from reboot regulation, start to cycle again allowing the cell cycle to continue. Our study might therefore help explain why cell cycle transitions can be regulated via a central oscillator which phase-locks independent oscillatory modules controlling specific cell-cycle events. This type of regulation readily allows for a regulatory mechanism by which defects in cell cycle transitions can cause the transition to be repeated, rather than being arrested, when defects occur.

It will be interesting to see if other cell types respond to cell cycle defects by imposing reboot-type regulation and if this type of regulation does in fact utilize the phase-locking model in which intrinsically autonomous regulatory cycles are locked at once-per-cell-cycle through entrainment by the Cdk oscillator. Analysis of mitotic regulators and how they might play roles in reboot regulation should provide fertile areas for further study.

## Supporting Information

S1 Fig Changes in nuclear structure during *A*. *nidulans* mitosis. (PDF)

S2 Fig Structure of Ima1. (PDF)

S1 Table *Aspergillus nidulans* strains used in this study. (PDF)
