# Supplementary material for: The Inner Nuclear Membrane Protein Src1 Is Required for Stable Post-Mitotic Progression into G1 in Aspergillus nidulans
Source: PLoS One. 2015 Jul 6;10(7):e0132489. doi: 10.1371/journal.pone.0132489 (PMC4492595; doi:10.1371/journal.pone.0132489)
Supplement: S1 Fig — (PDF) [file pone.0132489.s001.pdf]

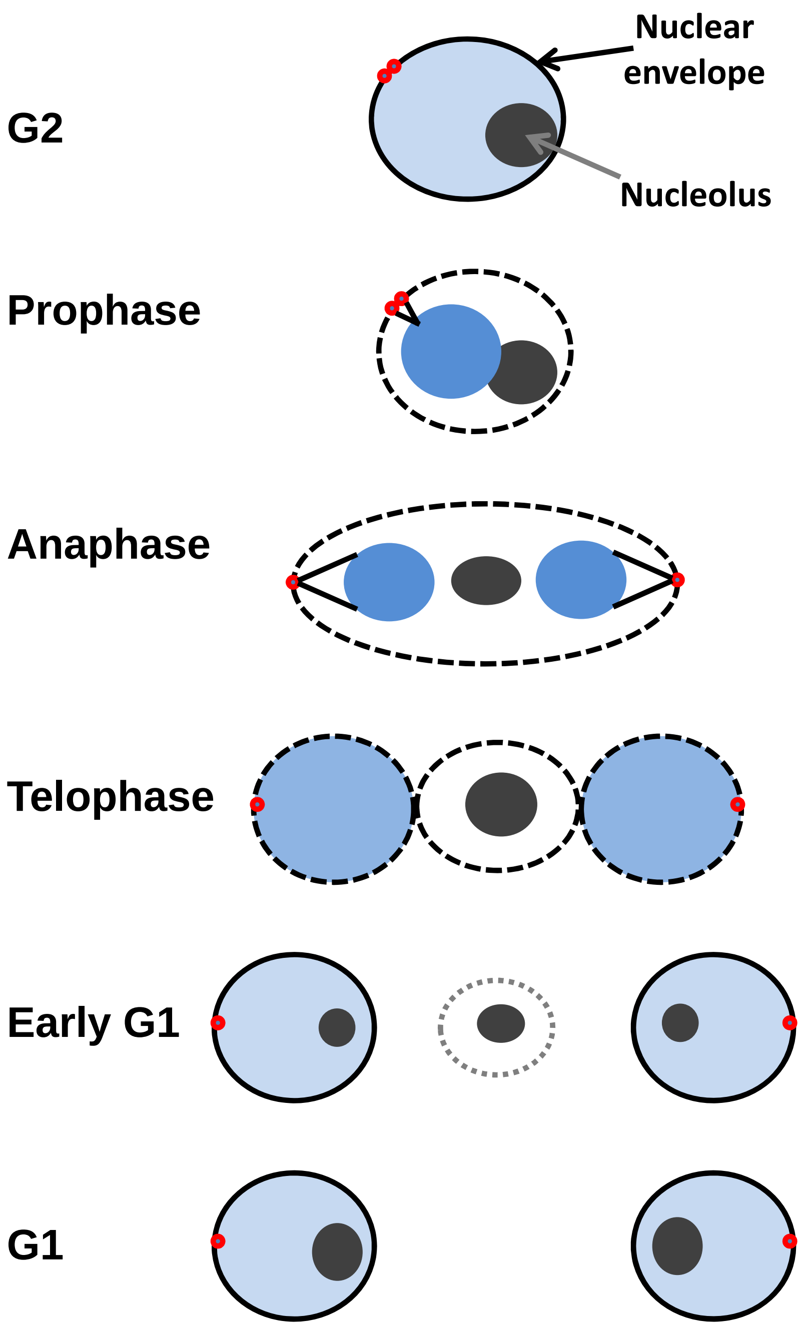

S1 Supplemental Figure. Changes in nuclear structure during *A. nidulans* mitosis. At **G2** DNA is surrounded by the nuclear envelope (solid black circle) in which the duplicated spindle pole bodies (red spheres) are embedded. As nuclei enter mitosis (**prophase**), nuclear pore complexes (NPCs) undergo partial disassembly which permeabilizes the NE (dotted black line) which allows nuclear proteins to escape into the cytoplasm. During prophase DNA also starts to condense (darker blue) and the SPBs seed early spindle formation. During **anaphase**, DNA is segregated into two which separates the nucleolus (black sphere) from DNA. As nuclei progress into **telophase** double restrictions of the NE result in exclusion of the nucleolus to the cytoplasm. As nuclei progress into **early G1** NPCs reassemble and nuclear transport is reestablished. Nucleolar proteins begin to disperse from the parental nucleolus and these released proteins are imported into daughter nuclei to form their nucleolus. DNA also decondenses. As nuclei finish their transition into **G1** all proteins of the parental nucleolus are re-imported into daughter nuclei to form a new nucleolus within each daughter G1 nucleus. The entire process takes on average 10-15 minutes.
