# Supplementary figures and images for: The Inner Nuclear Membrane Protein Src1 Is Required for Stable Post-Mitotic Progression into G1 in Aspergillus nidulans
Source: PLoS One. 2015 Jul 6;10(7):e0132489. doi: 10.1371/journal.pone.0132489 (PMC4492595; doi:10.1371/journal.pone.0132489)

S2 Supplemental Figure

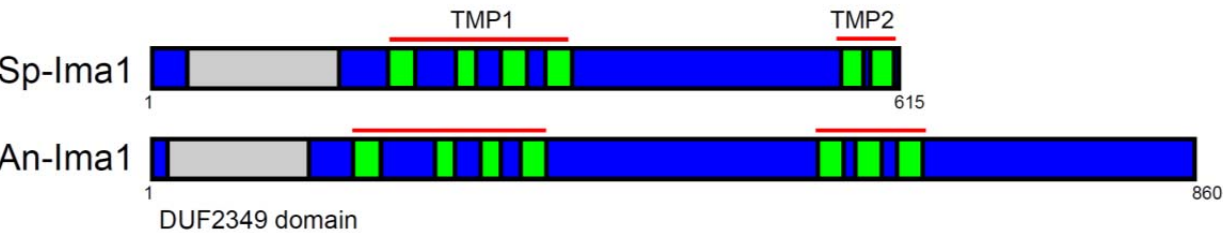

Supplement: S2 Fig — (PDF) [file pone.0132489.s002.pdf]
