# Supplementary material for: The Inner Nuclear Membrane Protein Src1 Is Required for Stable Post-Mitotic Progression into G1 in Aspergillus nidulans
Source: PLoS One. 2015 Jul 6;10(7):e0132489. doi: 10.1371/journal.pone.0132489 (PMC4492595; doi:10.1371/journal.pone.0132489)
Supplement: S1 Table — (PDF) [file pone.0132489.s003.pdf]

SI Table. *Aspergillus nidulans* strains used in this study

Strain	Genotype	
HH063	ΔAn-src1::pyrG^{AF}; An-Ima1-GFP::pyroA; nimT23; ΔyA::NLS-DsRed;	Transformation of HA449(heterokaryon)
HH069	ΔAn-src1::pyrG^{AF}; nimT23; An-Nup37-GFP::pyroA^{AF}; ΔyA::NLS-DsRed;	Transformation of HA349 (heterokaryon)
HH070	ΔAn-src1::pyrG^{AF}; Nup2-GFP::pyroA; nimT23; ΔyA::NLS-DsRed;	Transformation of HA377 (heterokaryon)
HH071	ΔAn-src1::pyrG^{AF}; nimT23; An-Nup49-CR::pyroA^{AF}; An-Ndc1-GFP::riboB^{AF};	Transformation of HA408 (heterokaryon)
HH072	ΔAn-src1::pyrG; Nup170-chRFP-pyroA^{AF}; Ndc1-GFP-riboB^{AF};	Transformation of HA424 (heterokaryon)
HH073	ΔAn-src1::riboB; cyclinB-GFP-pyrG^{AF}	Transformation of LO1400 (heterokaryon)
HH075	ΔAn-src1::pyrG; nimT23; tubA-GFP; An-H1-chRFP::pyroA^{AF}	Transformation of HA375 (heterokaryon)
HH080	ΔAn-src1::pyrG^{AF}; An-Bop1-GFP::riboB^{AF}; Nup49-chRFP::pyroA^{AF};	Transformation of CDS844 (heterokaryon)
HH081	ΔAn-src1::pyrG; cyclin B-GFP::pyroA^{AF}; Nup49-chRFP::pyroA^{AF};	Transformation of CDS851 (heterokaryon)
HH083	ΔAn-src1::riboB^{AF}; ΔyA::NLS-DsRed; tubA-GFP;	Transformation of HA347 (heterokaryon)
HH085	ΔAn-src1::pyrG^{AF}; An-Nup49-chRFP::pyroA^{AF}; An-Gcp3-GFP::riboB$^{AF;}$	Transformation of HA403 (heterokaryon)
HH087	ΔAn-src1::pyrG^{AF}; An-Nup49-GFP::riboB^{AF}; ΔyA::NLS-DsRed;	Transformation of SM112 (heterokaryon)
HA347	ΔyA::NLS-DsRed; tubA-GFP; (argB2?); riboB2; pyrG89;pyroA4; ΔnkuA::argB; chaA1	
HA349	nimT23; An-Nup37-GFP::pyroA^{AF}; ΔyA::NLS-DsRed; (argB2?; pyroA4); pyrG89; ΔnkuA::argB	
HA375	nimT23; tubA-GFP; An-H1-chRFP::pyroA^{AF}; pyrG89; (pyroA4; argB2?); ΔnkuA::argB	
HA377	Nup2-GFP::pyroA; nimT23; ΔyA::NLS-DsRed; pyrG89; (pyroA4; argB2?) ; ΔnkuA::argB	

HA408	*nimT23; An-Nup49-chRFP::pyroA*^{AF}*; An-Ndc1-GFP::riboB*^{AF}*; pyrG89; ΔnkuA::argB; wA3; (pyroA4; riboB2?; argB2*
HA424	*Nup170-chRFP-pyroA*^{AF}*; Ndc1-GFP-riboB*^{AF}*; ΔnkuA::argB; pyrG89; fwA1(argB2? pyroA4?; riboB2?)*
HA449	*An-Ima1-GFP::pyroA*^{AF}*; nimT23; ΔyA::NLS-DsRed; pyrG89; (pyroA4; argB2?) ; ΔnkuA::argB*
HA451	*An-Src1-GFP::pyroA; nimT23; ΔyA::NLS-DsRed; pyrG89; (pyroA4; argB2?) ; ΔnkuA::argB*
CDS844	*An-Bop1-GFP::riboB*^{AF}*; Nup49-CR::pyroA*^{AF}*; ΔnKuA::argB; pyrG89; pabaA1; pyroA4; wA3 (fwA1? riboB2? argB2?)*
CDS851	*cyclin B-GFP::pyroA*^{AF}*; Nup49-CR::pyroA*^{AF}*; ΔnKuA::argB; pyroA4; argB2; pyrG89; sE15; nirA14; wA3; fwA1; chaA1*
SM112	*An-Nup49-GFP::riboB*^{AF}*; ΔyA::NLS-DsRed; pyrG89*
LU223	*An-Src1-GFP-pyrG*^{AF}*; H1-mRFP-pyrG*^{AF} *(pyrG89); SE15?; nirA14?; wA3*
LU255	*An-Erg24-mRFP-pyroA*^{AF}*; An-Src1-GFP-pyrG*^{AF} *(pyrG89?); argB2; pyroA4; wA3; nirA14?*
